# A genomic instability-related lncRNA model for predicting prognosis and immune checkpoint inhibitor efficacy in breast cancer

**DOI:** 10.3389/fimmu.2022.929846

**Published:** 2022-08-05

**Authors:** Ying Jiao, Shiyu Li, Xuan Wang, Ming Yi, Hongqu Wei, Shanjie Rong, Kun Zheng, Li Zhang

**Affiliations:** ^1^ Department of Oncology, Tongji Hospital of Tongji Medical College, Huazhong University of Science and Technology, Wuhan, China; ^2^ Department of Anesthesiology, Tongji Hospital, Tongji Medical College, Huazhong University of Science and Technology, Wuhan, China; ^3^ Department of Radiology, The First Affiliated Hospital of Guangxi Medical University, Nanning, China; ^4^ The Center for Biomedical Research, Department of Respiratory and Critical Care Medicine, Key Laboratory of Pulmonary Diseases of Health Ministry, Tongji Hospital, Tongji Medical College, Huazhong University of Sciences and Technology, Wuhan, China

**Keywords:** genomic instability, lncRNAs, prognostic model, immune checkpoint, breast cancer, autoimmune diseases

## Abstract

Breast cancer has overtaken lung cancer as the most frequently diagnosed cancer type and is the leading cause of death for women worldwide. It has been demonstrated in published studies that long non-coding RNAs (lncRNAs) involved in genomic stability are closely associated with the progression of breast cancer, and remarkably, genomic stability has been shown to predict the response to immune checkpoint inhibitors (ICIs) in cancer therapy, especially colorectal cancer. Therefore, it is of interest to explore somatic mutator-derived lncRNAs in predicting the prognosis and ICI efficacy in breast cancer patients. In this study, the lncRNA expression data and somatic mutation data of breast cancer patients from The Cancer Genome Atlas (TCGA) were downloaded and analyzed thoroughly. Univariate and multivariate Cox proportional hazards analyses were used to generate the genomic instability-related lncRNAs in a training set, which was subsequently used to analyze a testing set and combination of the two sets. The qRT-PCR was conducted in both normal mammary and breast cancer cell lines. Furthermore, the Kaplan–Meier and receiver operating characteristic (ROC) curves were applied to validate the predictive effect in the three sets. Finally, the Cell-type Identification by Estimating Relative Subsets of RNA Transcripts (CIBERSORT) algorithm was used to evaluate the association between genomic instability-related lncRNAs and immune checkpoints. As a result, a six-genomic instability-related lncRNA signature (U62317.4, MAPT-AS1, AC115837.2, EGOT, SEMA3B-AS1, and HOTAIR) was identified as the independent prognostic risk model for breast cancer patients. Compared with the normal mammary cells, the qRT-PCR showed that HOTAIR was upregulated while MAPT-AS1, EGOT, and SEMA3B-AS1 were downregulated in breast cancer cells. The areas under the ROC curves at 3 and 5 years were 0.711 and 0.723, respectively. Moreover, the patients classified in the high-risk group by the prognostic model had abundant negative immune checkpoint molecules. In summary, this study suggested that the prognostic model comprising six genomic instability-related lncRNAs may provide survival prediction. It is necessary to identify patients who are suitable for ICIs to avoid severe immune-related adverse effects, especially autoimmune diseases. This model may predict the ICI efficacy, facilitating the identification of patients who may benefit from ICIs.

## Introduction

Breast cancer had overtaken lung cancer as the most frequently diagnosed cancer type and remained the leading cause of death for women worldwide by 2020 (1, 2). The most widely used classification of breast cancer is defined according to the expression of the progesterone receptor, estrogen receptor (ER), and human epidermal growth factor receptor 2 (HER2) (3). Moreover, breast cancer is a disease with high heterogeneity, resulting in challenges in treatment. Because of the high death rate, it is critical to identify novel prognostic biomarkers and develop suitable treatment plans for breast cancer patients.

Genomic instability (GI) refers to cells acquiring genomic alterations at an increased rate, which is divided into small structural variations and significant structural variations (4). It is reported that GI can be deemed a hallmark of cancer development due to the enhanced survival ability of cancer cells (5, 6). Moreover, the mechanism underlying increased GI involves the failure of DNA damage repair, DNA replication stress, transcription-associated stress, and chromothripsis (7). Notably, the immune checkpoint inhibitors (ICIs) have achieved unprecedented success in microsatellite instability-high (MSI-H)/deficient mismatch repair (dMMR) colorectal cancer (8). Meanwhile, programmed cell death protein 1 (PD-1) blockade has become a first-line treatment option for MSI-H/dMMR metastatic colorectal cancer as recommended in the guideline (9). Thus, it is suggested that GI may be closely associated with immune checkpoint blockade treatment. However, nearly half of the patients receiving immunotherapy are refractory to ICIs (10), and there are few biological predictive factors to stratify the patients who receive the ICI therapy, which can be a novel research direction for GI. Recently, efforts to further understand GI in breast cancer have also been focused on its roles in tumor initiation, progression, and, particularly, prognostic prediction.

Long non-coding RNAs (lncRNAs) are transcripts that include more than 200 nucleotides without the potential of coding proteins (11). During the past decades, numerous lncRNAs were identified to be aberrantly expressed in manifold cancers owing to the rapid development of next-generation sequencing technologies, and the roles of lncRNAs in the different biological processes have been realized gradually (12, 13). Emerging studies showed a noticeable link between lncRNAs and genomic stability (14, 15). The most well-known example is the non-coding RNA activated by DNA damage (NORAD, also termed as LINC00657), which can maintain genomic stability *via* sequestering pumilio RNA binding family member 1 proteins (16). Another study revealed that the interaction between NORAD and RNA binding motif protein X-linked, a component of the DNA-damage response, contributed to the maintenance of genomic stability (17). Although abundant studies have verified the connection of lncRNAs with genomic stability, the roles of GI-associated lncRNAs and their clinical value remain to be further investigated.

At present, lncRNAs are considered as an independent prognostic biomarker in cancer (18), such as HOX transcript antisense RNA (HOTAIR) in ER^+^ breast cancer (19). Nonetheless, a single lncRNA as the predictive biomarker is not gratifying, due to tremendous false-positive and -negative results (20). Here, we analyzed the lncRNA expression and somatic mutation data in breast cancer from The Cancer Genome Atlas (TCGA) and developed a six-mutator-derived lncRNA signature to reflect GI and predict the survival prognosis for breast cancer patients.

## Materials and methods

### Data source

The RNA-seq data, somatic mutation features, and clinical information of breast cancer patients were acquired from the TCGA database (https://portal.gdc.cancer.gov/). Then, the RNA-seq data were divided into lncRNA and mRNA expression profiles. A total of 1,109 patients with breast cancer were included in the study to identify the lncRNA-related prognostic model. Moreover, the prognostic value of these lncRNAs was validated in an interactive web, Gene Expression Profiling Interactive Analysis (GEPIA, http://gepia.cancer-pku.cn/detail.php?gene=&clicktag=survival) (21). GEPIA included data from TCGA and the Genotype-Tissue Expression (GTEx) projects. TCGA and GEPIA are open public databases, and there was no need for ethics approval in the study.

### Identification of GI-related lncRNAs

To begin with, the mutation count of each patient was calculated and ranked by analyzing somatic mutation profiles from TCGA (https://portal.gdc.cancer.gov/). The top 25% of patients were assigned as the genomically unstable (GU) group, while the last 25% were defined as the genomically stable (GS) group (22). Secondly, differentially expressed lncRNAs were identified by analyzing the lncRNA expression differences between the GU and GS groups with the Wilcoxon test. GI-related lncRNAs were defined when the |log fold change| (logFC) > 1 and the false discovery rate (FDR)-adjusted *p*< 0.05.

### Functional enrichment analysis

All the patients with lncRNA expression data were divided into a GU-like or a GS-like group using genome instability-related lncRNAs and conducting hierarchical cluster analyses. The somatic mutation count and the expression of some immune checkpoints, including PD-1, programmed cell death receptor ligand 1 (PD-L1), indoleamine 2,3-dioxygenase 1 (IDO1), and tryptophan 2,3-dioxygenase 2 (TDO2), between the two groups, were determined. Furthermore, the correlation test between the genome instability-related lncRNAs and mRNA expression was conducted to get the Pearson correlation coefficients. The paired top 10 mRNAs were regarded as co-expressed followers of each GI-related lncRNA. To discover the potential function of these lncRNAs, we screened related protein-coding genes and performed Gene Ontology (GO) analysis and Kyoto Encyclopedia of Genes and Genomes (KEGG) enrichment analysis (23).

### Definition of the GI-related lncRNA prognostic model

All breast cancer patients were defined as the TCGA set and were also divided into two sets randomly, including a training set and a testing set. We performed the Chi-square test to evaluate the association of each set with other critical clinical characteristics. Subsequently, univariate and multivariate analysis by Cox proportional hazards regression model was used to evaluate the link between the expression of GI-related lncRNAs and prognosis in breast cancer patients in the training set. After univariate Cox regression analysis, the survival-related lncRNAs were shown as the forest plot when the *p*-value was<0.05, in which hazard ratio (HR) and 95% confidence interval (CI) were calculated with the survival and survminer package in R. After multivariate analysis, the prognostic risk model independent of other clinical features was built. According to the expression and coefficients of the GI-related lncRNAs and patient survival, the formula of an lncRNA-based prognostic risk score for a breast cancer patient was defined as follows:


$$\text{Risk score}=\sum_{i=1}^{n}\text{expression}\left({\text{lncRNA}}_{i}\right)$$

*Coefficient (lncRNA*_i_*)

Firstly, the risk score of each patient in the training set was computed. Then, the median risk score of patients was regarded as the cutoff value. On the basis of the cutoff value of the training set, the patients in the training set, the testing set, and the TCGA set were categorized into high- or low-risk groups separately. Finally, the testing set along with the TCGA set was used to verify the feasibility of the prognostic risk model acquired from the data of the training set.

### Validation of the GI-related lncRNA prognostic model

Survival curves were plotted in the training set, the testing set, and the TCGA set to validate the predictive ability of the risk score, in which the log-rank test was performed with a *p<* 0.05 as statistical significance. The receiver operating characteristic (ROC) curves with 3 and 5 years were used to test the performance of the lncRNA-related prognostic model, which showed the sensitivity and specificity. Furthermore, the association between the risk score and the expression of each lncRNA in the prognostic model was investigated in the three sets. Likewise, the relations between the risk score and somatic mutation level, and the expression level of IDO1 as well as TDO2 were explored. Then, the prognostic lncRNAs were validated in GEO datasets with breast cancer patients. The landscape profiling of somatic gene mutations in the high- or low-risk group from the TCGA was conducted as a waterfall plot with the Maftools package in the R software. Moreover, stratification analysis of the prognostic risk model by age, stage, and gender was estimated using the univariate Cox analysis and the log-rank test. Finally, the prognostic lncRNA signature was compared with other signatures published in existing studies.

### The relation between the risk score and immune function

The Cell-type Identification by Estimating Relative Subsets of RNA Transcripts (CIBERSORT) algorithm (24) was applied to evaluate the immune-related signature of each patient with breast cancer. The expression of immune checkpoints was analyzed to identify the association of lncRNA-related risk score with cancer immunity. To determine the difference of signaling pathways between the high- and low-risk groups, multiple gene set enrichment analysis (GSEA) was performed through the GSEA software (4.1.0) and R packages (25).

### Cell culture

Normal mammary epithelial cells HBL100 as well as five human breast cancer cell lines MDA-MB-231, MDA-MB-468, Sum159, H578T, and SKBR3 were obtained from the Department of Oncology (Tongji Hospital, Wuhan, China) and cultured in Dulbecco’s modified Eagle medium (DMEM, Hyclone), which contained 10% fetal bovine serum (FBS). Cells were incubated in an incubator containing 5% CO_2_ at 37°C.

### RNA extraction and real-time PCR assay

Total RNA was extracted using Trizol reagent (Invitrogen, Carlsbad, USA) and the manufacturer’s manual was followed. Complementary DNA for reverse transcription was synthesized by the Prime Script RT kit (Takara, Tokyo, Japan). Real-time PCR analysis was then performed. The 2^−ΔΔ^Ct method was applied to determine differences between multiple samples. The primer sequences are as follows. HOTAIR primer sequences: forward strand, 5′-ACTCTGACTCGCCTGTGCTCTG-3′; reverse strand, 5′-AGTGCCTGGTGCTCTCTTACCC-3′; SEMA3B-AS1 primer sequences: forward strand, 5′-GTCCTGAAGCTGAGTCTGGTGAAC-3′; reverse strand, 5′-CTCCACTCTGCCACTGTCAACATAC-3′; EGOT primer sequences: forward strand, 5′-TAACGCACTAGAGGAGACAGAGACG-3′; reverse strand, 5′-GTTGCTAGTTGGACAGTCGGTATGG-3′; MAPT-AS1 primer sequences: forward strand, 5′-CGGAACCAGAAGGGAGGGATTTG-3′; reverse strand, 5′-CACAGAGACACACAGGGAGAATGC-3′.

### Statistical analysis

Statistical analysis was conducted with Perl version 5.18.4 (https://www.perl.org/) and R version 4.0.3 (Package: limma, pheatmap, sparcl, ggpubr, clusterProfiler, org.Hs.eg.db, enrichplot, ggplots, survival, caret, glmnet, survminer, timeROC, e1071, parallel, preprocessCore, plyr, grid, gridExtra, and maftools). GSEA was performed for functional annotation. The real-time PCR data were analyzed with the GraphPad Prism 8.0 software and the two-sample *t*-test. Two-tailed *p*< 0.05 was considered as statistically significant (*p < 0.05, **p < 0.01, ***p < 0.001).

## Results

### Identification of GI-related lncRNAs

**Figure 1:**
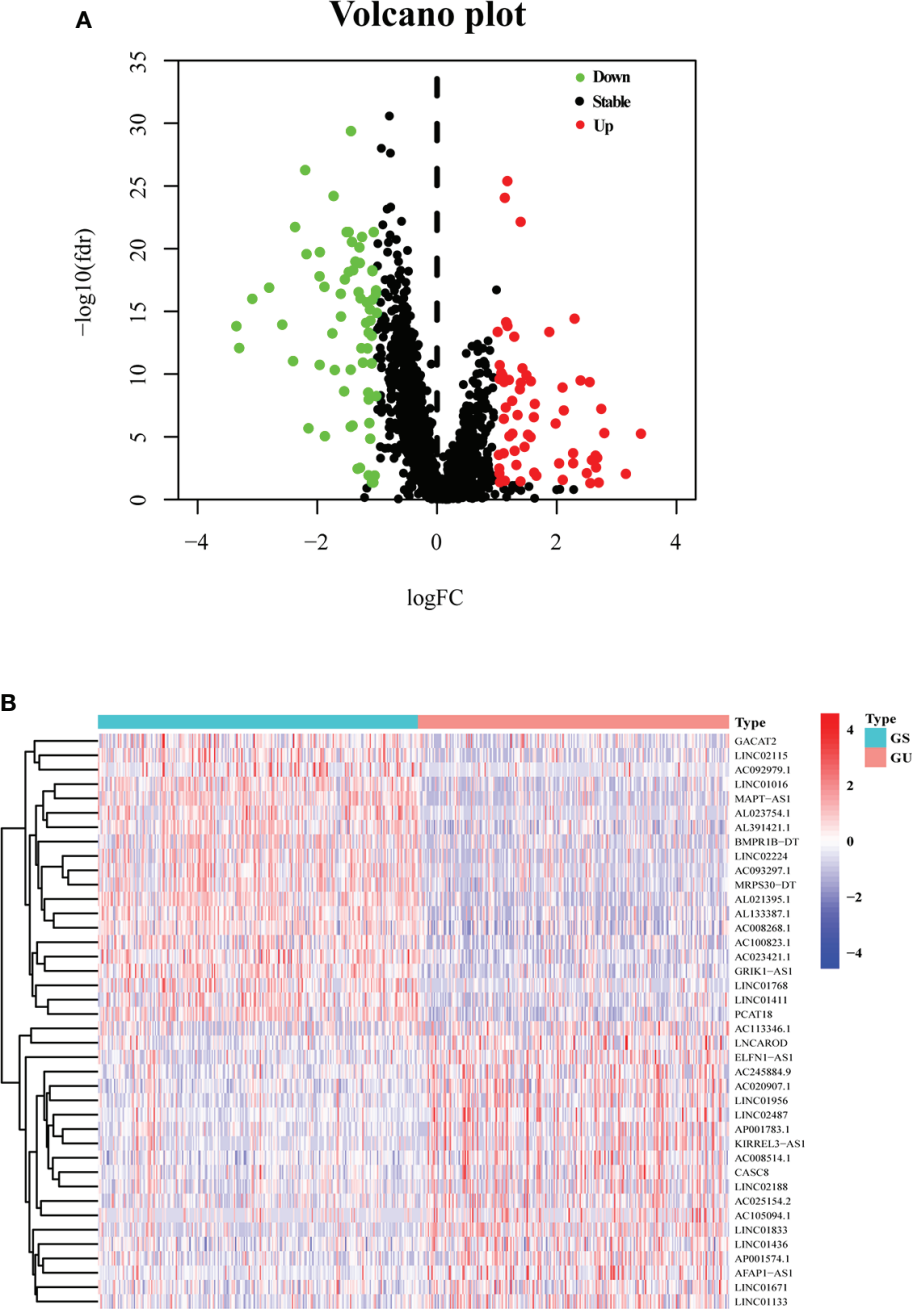
One hundred twenty-eight GI-related lncRNAs in breast cancer from TCGA. **(A)** A total of 128 GI-related lncRNAs are shown in a volcano plot. Sixty-three were upregulated and shown in red. Sixty-five were downregulated and shown in green. **(B)** Heatmap of the top 20 upregulated and top 20 downregulated GI-related lncRNAs. The top 25% (*n* = 252) and the last 25% (*n* = 259) mutated patients were selected as GU and GS. The green and red bars represent GU and GS, respectively. Red represents upregulated lncRNA, and blue denotes downregulated lncRNA.

After calculating the somatic mutation count of each breast cancer patient, the top 25% (*n* = 252) and the last 25% (*n* = 259) of the patients were grouped as GU and GS, respectively. Subsequently, 1,833 differentially expressed lncRNAs between GU and GS were identified by analyzing the lncRNA expression data with the Wilcoxon–Mann–Whitney test. Based on the criteria of |logFC| > 1 and FDR< 0.05, 128 differentially expressed lncRNAs were identified as GI-related lncRNAs in breast cancer, in which 63 were upregulated and 65 were downregulated. Then, a volcano plot was produced to show the 128 GI-related lncRNAs (**Figure 1A**), and a heatmap was used to demonstrate the differential expression of the top 20 upregulated and 20 downregulated GI-related lncRNAs (**Figure 1B**).

### Analysis of GI-related lncRNAs between the GS-like and GU-like groups

**Figure 2:**
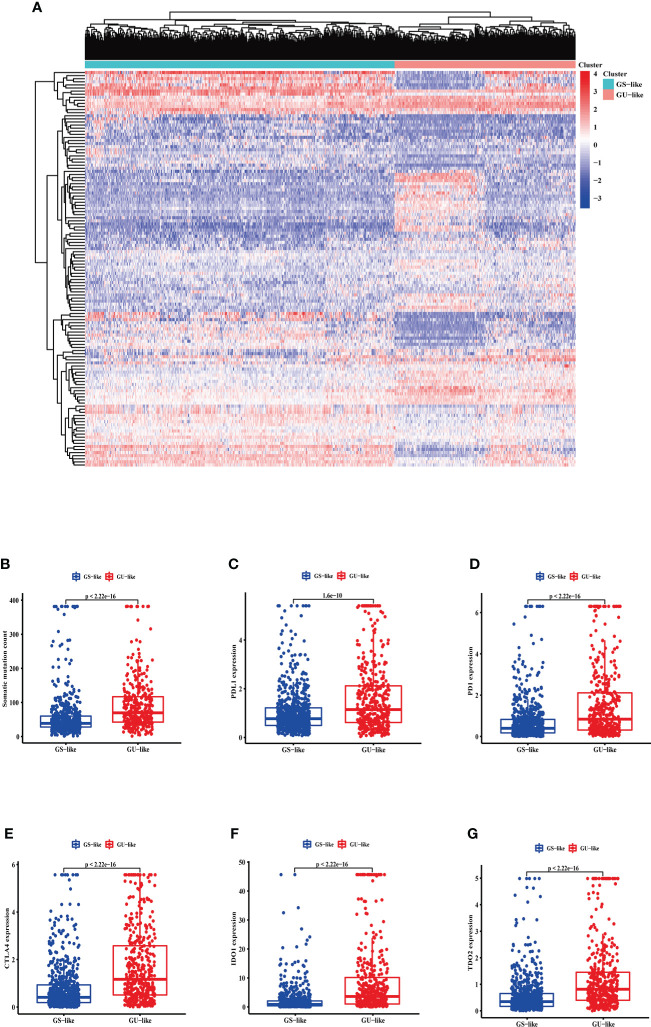
The somatic mutations and the expression level of some pivotal immune checkpoints in the GS-like and GU-like group. **(A)** Unsupervised clustering of 1,109 breast cancer patients according to the expression levels of 128 genomic instability-related lncRNAs. The right red cluster is the GU-like group, and the left blue cluster is the GS-like group. **(B)** Boxplots of somatic cumulative mutations in the GU-like and GS-like groups. The somatic mutation counts in the GU-like group are higher than in the GS-like group. The expression level of PDL1 **(C)**, PD1 **(D)**, CTLA4 **(E)**, IDO1 **(F)**, and TDO2 **(G)** in the GU-like group are higher than the GS-like group. Horizontal lines are median values. GS, genomically stable group; GU, genomically unstable group.

LncRNA expression profiles with 1,109 breast cancer patients were analyzed by unsupervised hierarchical clustering using the 128 GI-related lncRNAs. Then, the 1,109 samples were clustered into the GS-like group (*n* = 700) and GU-like group (*n* = 409) (**Figure 2A**). The GU-like group had a higher somatic mutation count than the GS-like group (*p*< 0.001, **Figure 2B**).

Moreover, given the potential relation between GI and immune checkpoints, the mRNA levels of PD-L1 (**Figure 2C**), PD-1 (**Figure 2D**), cytotoxic T-lymphocyte-associated protein 4 (CTLA4) (**Figure 2E**), IDO1 (**Figure 2F**), and TDO2 (**Figure 2G**) between the GS-like and GU-like groups were compared. The result indicated that the GU-like group had a significantly higher expression level of the five immune checkpoints mentioned above.

**Figure 3:**
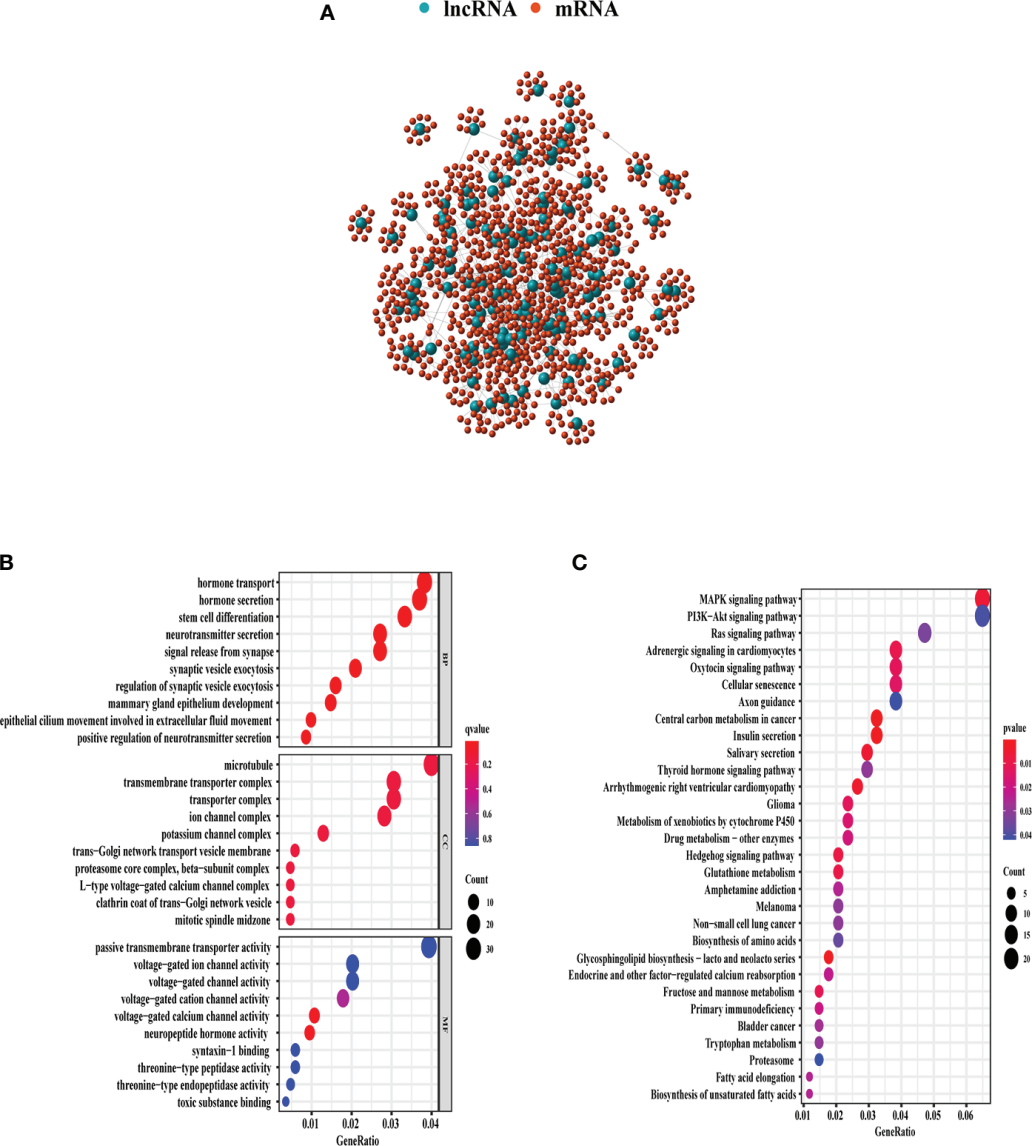
GO and KEGG enrichment analyses of 1,280 genes related with 128 lncRNAs demonstrated in the bubble plot. **(A)** The 1,280 genes related to 128 lncRNAs. **(B)** The top 10 enriched terms of BP, CC, and MF in GO analysis. **(C)** The top 30 enriched terms in KEGG analysis. The bubble size shows the count of related genes enriched under each term. GO, gene ontology; BP, biological process; CC, cellular component; MF, molecular function; KEGG, Kyoto Encyclopedia of Genes and Genomes.

Through Spearman’s correlation analysis, the top 10 protein-coding genes were chosen for each GI-related lncRNA, which produced 1,280 genes in all (**Figure 3A**). The enriched GO terms and KEGG pathways were analyzed for the 1,280 genes. In the biological process terms of GO, our analysis indicated that most protein-coding genes were enriched in “hormone transport” (GO:0009914), “hormone secretion” (GO:0046879), and “stem cell differentiation” (GO:000048863) (**Figure 3B**). In the KEGG analysis, our result showed that these genes were enriched in the “MAPK signaling pathway” (hsa04010) and “PI3K−Akt signaling pathway” (hsa04151) (**Figure 3C**).

### Identification of 6-GI-related lncRNA prognostic signature for breast cancer

**Figure 4:**
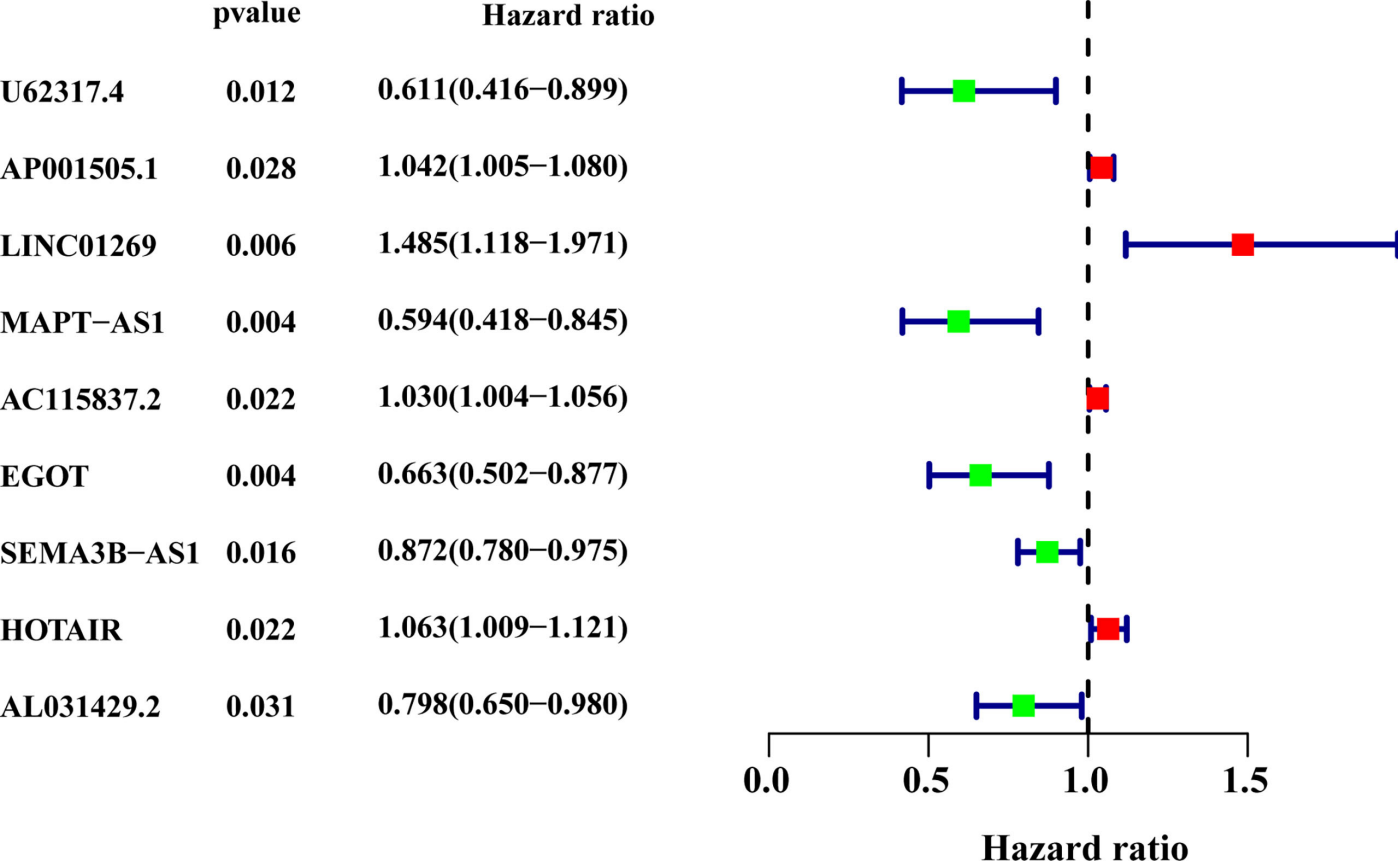
The forest plot of the nine lncRNAs generated from the univariate Cox regression analysis (criteria: *p*-value < 0.05). HR, hazard ratio; CI, confidence interval.

All patients with survival information were divided into a training set with 520 patients and a testing set with 519 patients at random. As shown in **Table 1**, there was no correlation between the two groups in the demographic, clinical, or pathological features as shown in the *χ* ^2^ test. Using the 128 lncRNA expression levels in 1,039 breast cancer patients with survival information, we further investigated survival-related lncRNAs with univariate Cox proportional hazard regression analysis in the training set. We found that nine lncRNAs were markedly correlated with the prognosis of breast cancer patients (**Figure 4**). Then, multivariate proportional hazards (Cox) regression analysis was conducted to identify the independent prognostic model using the nine lncRNA expression levels and demographic and clinical features, including age, gender, and stage. Finally, six of the nine candidate lncRNAs [U62317.4, MAPT antisense RNA 1 (MAPT-AS1), AC115837.2, glutathione reductase and glutamic oxaloacetic transaminase (EGOT), Semaphorin 3B antisense RNA 1 (SEMA3B-AS1), and HOTAIR] were identified as the independent prognostic signature for breast cancer patients (**Table 2**). According to the coefficients and the expression of the six lncRNAs, the mutator-related lncRNA prognostic signature was defined as follows: risk score = (−0.6608 × expression level of U62317.4) + (−0.5443 × expression level of MAPT-AS1) + (0.0295 × expression level of AC115837.2) + (−0.2304 × expression level of EGOT) + (−0.1102 × expression level of SEMA3B-AS1) + (0.0529 × expression level of HOTAIR). The formula could evaluate the risk score and prognosis of breast cancer patients. In these lncRNAs, the coefficients of AC115837.2 and HOTAIR were positive, which showed that they were implicated in poor survival. However, the U62317.4, MAPT-AS1, EGOT, and SEMA3B-AS1 had negative coefficients associated with a good prognosis. Based on the prognostic signature consisting of the six mutator-related lncRNAs, the risk score of each patient in the training set, testing set, and the TCGA set was computed. The median risk score of the training set (1.557) was used as the cutoff value to divide the breast cancer patients in every set into high or low risk.

**Table 1:** The correlation between the two groups in demographic and clinical characteristics for breast cancer patients.

Covariates	Type	Total (*n* = 1,039)	Test (*n* = 519)	Train (*n* = 520)	*p*-value
Age	≤65	746 (71.8%)	366 (70.52%)	380 (73.08%)	0.3971
Age	>65	293 (28.2%)	153 (29.48%)	140 (26.92%)	
Gender	Female	1027 (98.85%)	515 (99.23%)	512 (98.46%)	0.3855
Gender	Male	12 (1.15%)	4 (0.77%)	8 (1.54%)	
Stage	Stage I–II	767 (73.82%)	374 (72.06%)	393 (75.58%)	0.2091
Stage	Stage III–IV	250 (24.06%)	134 (25.82%)	116 (22.31%)	
Stage	Unknown	22 (2.12%)	11 (2.12%)	11 (2.12%)	
T	T1–2	871 (83.83%)	425 (81.89%)	446 (85.77%)	0.0895
T	T3–4	165 (15.88%)	93 (17.92%)	72 (13.85%)	
T	Unknown	3 (0.29%)	1 (0.19%)	2 (0.38%)	
M	M0	862 (82.96%)	430 (82.85%)	432 (83.08%)	0.3972
M	M1	21 (2.02%)	8 (1.54%)	13 (2.5%)	
M	Unknown	156 (15.01%)	81 (15.61%)	75 (14.42%)	
N	N0	485 (46.68%)	237 (45.66%)	248 (47.69%)	0.6118
N	N1–3	537 (51.68%)	272 (52.41%)	265 (50.96%)	
N	Unknown	17 (1.64%)	10 (1.93%)	7 (1.35%)	

Chi-square test was used.

**Table 2:** The expression of the six lncRNAs generated from the multivariate Cox regression analysis.

Ensemble ID	Gene symbol	Strand	Genomic location	Coefficient	HR	95% CI	*p*-value
ENSG00000273272	U62317.4	+	chr22: 50,541,414-50,543,013	−0.660753711	0.516	0.335–0.796	0.003
ENSG00000264589	MAPT-AS1	–	chr17: 45,799,390-45,895,630	−0.544291478	0.580	0.395–0.852	0.005
ENSG00000235947	EGOT	–	chr3: 4,749,192-4,751,590	−0.230389874	0.794	0.618–1.021	0.073
ENSG00000232352	SEMA3B-AS1	–	chr3: 50,266,641-50,267,371	−0.110195358	0.896	0.801–1.002	0.054
ENSG00000228630	HOTAIR	–	chr12: 53,962,308-53,974,956	0.052897129	1.054	1.001–1.111	0.048
ENSG00000254080	AC115837.2	–	chr8: 74,609,698-74,633,320	0.029465848	1.030	1.006–1.054	0.013

CI, confidence interval; HR, hazard ratio; lncRNAs, long non-coding RNAs.

### Validation of the 6-GI-related lncRNA prognostic model

**Figure 5:**
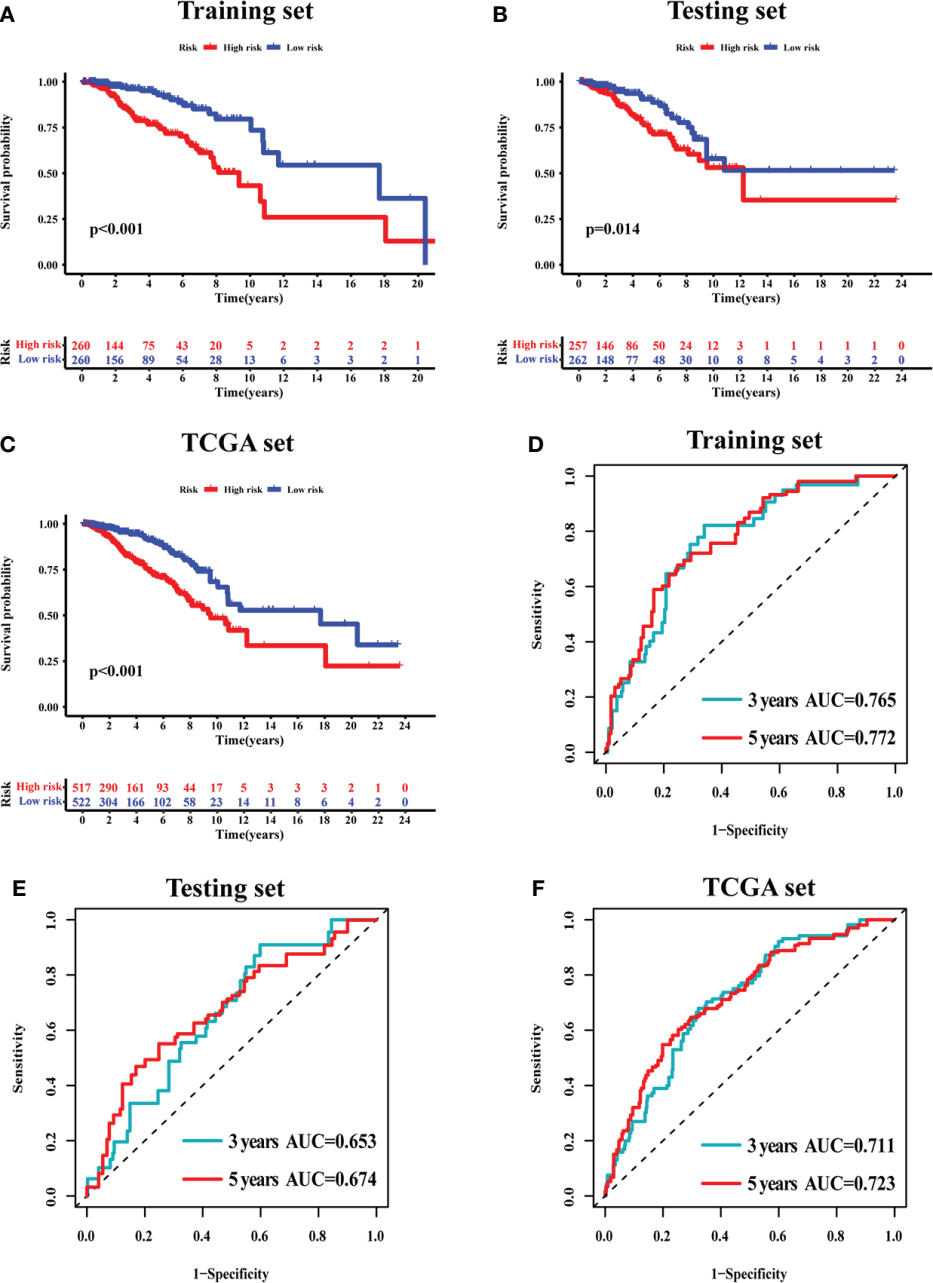
The prognostic value of the 6-GI-related lncRNA prognostic model in breast cancer patients. Overall survival was estimated by Kaplan–Meier for patients with a low or high risk predicted by the lncRNA-related model in the training set **(A)**, testing set **(B)**, and TCGA set **(C)**. Time-dependent ROC curves analysis of the lncRNA-related model was performed for 3-year and 5-year overall survival in the training set **(D)**, testing set **(E)**, and TCGA set **(F)**. AUC, area under ROC curve; ROC, receiver operating characteristic; TCGA, The Cancer Genome Atlas.

**Figure 6:**
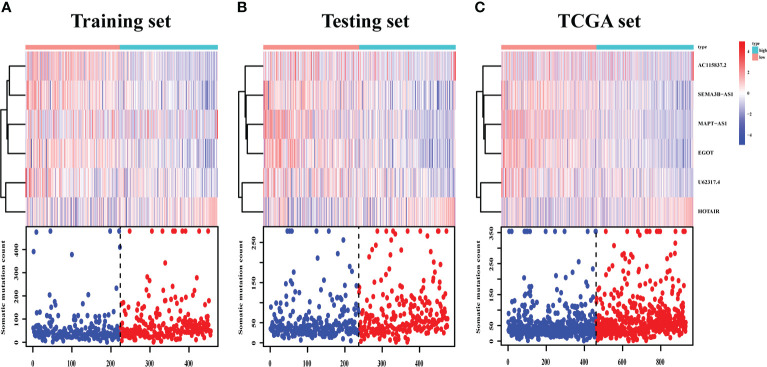
LncRNA expression and IDO1/TDO2 expression difference between patients with a high and low risk. LncRNA expression patterns and the distribution of somatic mutation counts in the training set **(A)**, testing set **(B)**, and TCGA set **(C)** with increasing risk score. TCGA, The Cancer Genome Atlas.

The survival analysis in the training set (**Figure 5A**), testing set (**Figure 5B**), and TCGA set (**Figure 5C**) suggested that the patients with a high risk had poorer survival rates than those with a low risk (log-rank test, *p*< 0.05). The time-dependent ROC curve analysis was conducted in the training set, and the result showed that the area under the ROC curve (AUC) for 3-year and 5-year overall survival (OS) was 0.765 and 0.772, respectively (**Figure 5D**), showing a high sensitivity and specificity of the GI-related lncRNA prognostic signature. Furthermore, the AUC for 3-year OS in the testing set was 0.653 and that for 5-year OS was 0.674 (**Figure 5E**), while the AUC for 3-year OS in the TCGA set was 0.711 and was 0.723 for 5-year OS (**Figure 5F**). On the basis of the risk score, we stratified the patients in the training set (**Figure 6A**), testing set (**Figure 6B**), and TCGA set (**Figure 6C**), and demonstrated the expression levels of the six GI-related lncRNAs and the somatic mutation counts. With the risk score increasing, the expression levels of AC115837.2 and HOTAIR were upregulated, while U62317.4, MAPT-AS1, EGOT, and SEMA3B-AS1 were downregulated.

**Figure 7:**
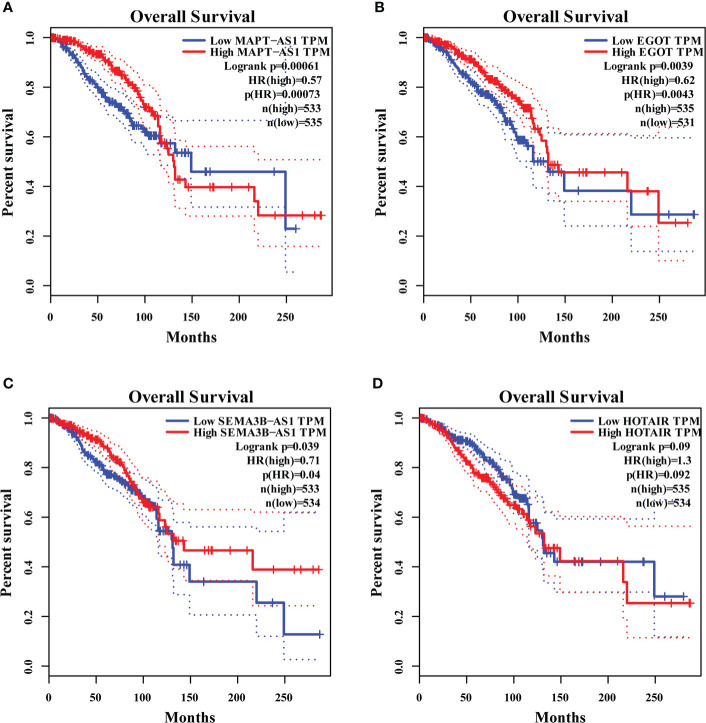
The overall survival analyses of MAPT-AS1, EGOT, SEMA3B-AS1, and HOTAIR on the online web, GEPIA. The high expression of MAPT-AS1 **(A)**, EGOT **(B)**, and SEMA3B-AS1 **(C)** predicts favorable survival, while the low HOTAIR **(D)** indicates favorable survival. GEPIA, gene expression profiling interactive analysis; TPM, transcripts per million.

We found that MAPT-AS1, EGOT, SEMA3B-AS1, and HOTAIR in the six GI-related lncRNAs were covered by GEPIA. The survival prediction of these lncRNAs was performed separately. The results indicated that the high MAPT-AS1 expression was significantly associated with a longer OS (log-rank test, *p*< 0.001, **Figure 7A**), and so were EGOT (log-rank test, *p*< 0.001, **Figure 7B**) and SEMA3B-AS1 (log-rank test, *p*< 0.001, **Figure 7C**). However, high HOTAIR expression showed no significant association with a poorer OS (log-rank test, *p* = 0.09, **Figure 7D**).

**Figure 8:**
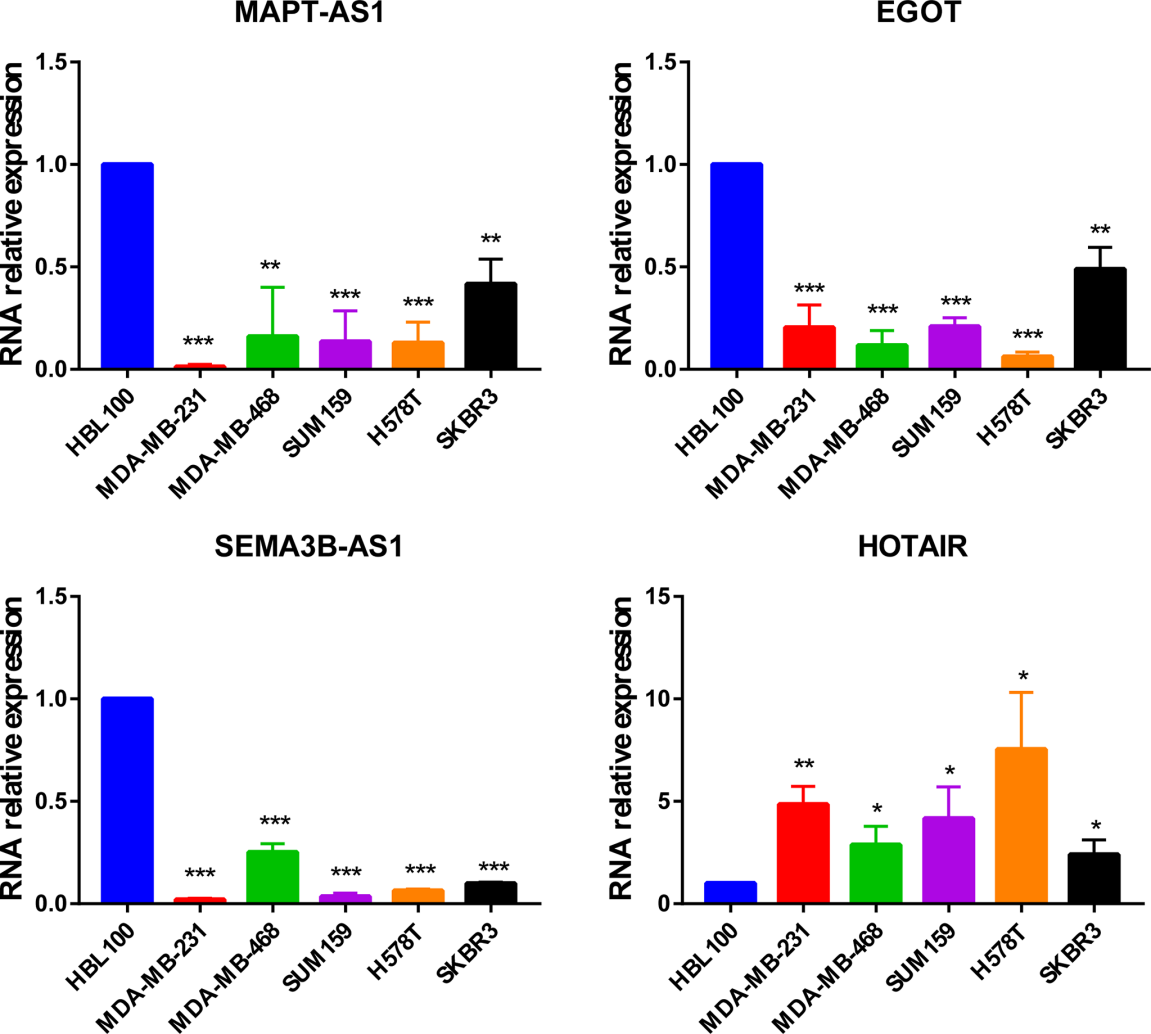
The expression of MAPT-AS1, EGOT, SEMA3B-AS1, and HOTAIR in human normal mammary epithelial cells and breast cancer cell lines. *p < 0.05, **p < 0.01, ***p < 0.001.

In addition, MAPT-AS1, EGOT, SEMA3B-AS1, and HOTAIR were further verified in normal mammary epithelial cells (HBL100) as well as five human breast cancer cell lines by real-time PCR. The results indicated that MAPT-AS1, EGOT, and SEMA3B-AS1 were downregulated while the expression of HOTAIR was increased in breast cancer cell lines, which were consistent with the predicted results (**Figure 8**).

### Landscape profile of somatic gene mutations

We obtained the somatic mutation profiles of 467 patients in the high-risk group and 459 patients in the low-risk group in the TCGA database. Most of the breast cancer patients had somatic mutations, with 85.87% (401/467) and 83.01% (381/459) in the high-risk group and low-risk group, respectively. The waterfall plot demonstrated the top 20 mutated genes in the patients in the high-risk group (**Supplementary Figure 1A**) and low-risk group (**Supplementary Figure 1B**). We found that the most frequently mutated gene in the high-risk group was TP53 (50%), while that in the low-risk group was PIK3CA (37%). In most cases, there was more mutability for each gene in the high-risk group. Furthermore, the most frequent gene alteration type was missense mutation.

### Independent prognostic value of the 6-GI-related-lncRNA signature

The independence of the 6-GI-related-lncRNA signature from other clinical characteristics, including age, gender, and stage, was investigated by adopting univariate and multivariable Cox proportional hazards regression analysis. The risk score was significantly associated with OS and could be regarded as an independent prognostic predictor in each patient set (*p*< 0.01, **Table 3**). Besides risk score, both age and stage were independent factors and significantly associated with OS. To further investigate whether the prognostic model had a broad sphere of application, we sorted the patients according to their clinical features and observed the survival difference between the patients in the high- and low-risk groups. All the patients were divided into older patients with age > 65 and younger patients with age ≤ 65, female and male, and patients with stage I–II and patients with stage III–IV. In each group, patients were further stratified into high or low risk according to the median risk score. Our results suggested that patients with age > 65 and a high-risk score tended to have a poorer OS (log-rank test *p* = 0.012; **Supplementary Figure 2A**), and so were patients with age ≤ 65 and a high-risk score (log-rank test, *p*< 0.01; **Supplementary Figure 2B**). There was a significant association between the low-risk score and better OS in female patients (log-rank test *p*< 0.001; **Supplementary Figure 2C**), which was not observed in male patients (log-rank test *p* = 0.102; **Supplementary Figure 2D**). In patients with stage I–II (log-rank test *p*< 0.001; **Supplementary Figure 2E**) and stage III–IV (log-rank test *p* = 0.042; **Supplementary Figure 2F**), the higher-risk score predicted poorer OS. Moreover, our results showed that the prognostic model could be adapted in patients with T, N, and M stages (**Supplementary Figure 3**).

**Table 3:** Univariate and multivariate Cox regression analyses in the training, test, and TCGA sets.

Group	Variables	Univariable analysis			Multivariable analysis		
		HR	95% CI of HR	*p*-value	HR	95% CI of HR	*p*-value
Training set (*n* = 520)	Age	1.035	1.016–1.055	<0.001	1.042	1.021–1.064	<0.001
	Gender	1.173	0.162–8.479	0.874			
	Stage	2.068	1.539–2.779	<0.001	2.025	1.505–2.724	<0.001
	Risk Score	1.262	1.178–1.352	<0.001	1.206	1.124–1.293	<0.001
Testing set (*n* = 519)	Age	1.033	1.014–1.054	0.001	1.030	1.010–1.050	<0.001
	Gender	0.000	0–inf	0.996			
	Stage	2.255	1.581–3.217	<0.001	2.122	1.500–3.003	<0.001
	Risk Score	1.092	1.024–1.164	0.007	1.086	1.014–1.163	0.018
All patient set (*n* = 1,039)	Age	1.035	1.021–1.049	<0.001	1.036	1.022–1.050	<0.001
	Gender	0.852	0.119–6.104	<0.001			
	Stage	2.189	1.742–2.751	<0.001	2.142	1.717–2.673	<0.001
	Risk Score	1.128	1.086–1.173	<0.001	1.118	1.072–1.165	<0.001

CI, confidence interval; HR, hazard ratio.

### Analysis and comparison of the 6-GI-related lncRNA signature with other prognostic models in breast cancer

The effect of survival prediction was compared between our six-lncRNA signature (from now on referred to as JiaolncSig) and two other prognostic lncRNA models, the immune-related (referred to as LiulncSig) (26) and stemness-related signature (referred to as LilncSig) (27) in the same TCGA database with breast cancer patients. The AUC of JiaolncSig for 3-year OS was 0.711, while the AUC of LilncSig was 0.708 and 0.608 for LiulncSig (**Supplementary Figure 4A**). As for the AUC for 5-year OS, JiaolncSig (0.723) was also superior to the other two models (**Supplementary Figure 4B**).

Moreover, the JiaolncSig only included 6 lncRNAs, which was fewer than LilncSig (12 lncRNAs) and LiulncSig (7 lncRNAs). These results suggested our signature to be a better lncRNA-related prognostic model than the other two existing lncRNA signatures in breast cancer with more potential in clinical applications.

### Association between the 6-GI-related lncRNA signature and the immune checkpoints in breast cancer

**Figure 9:**
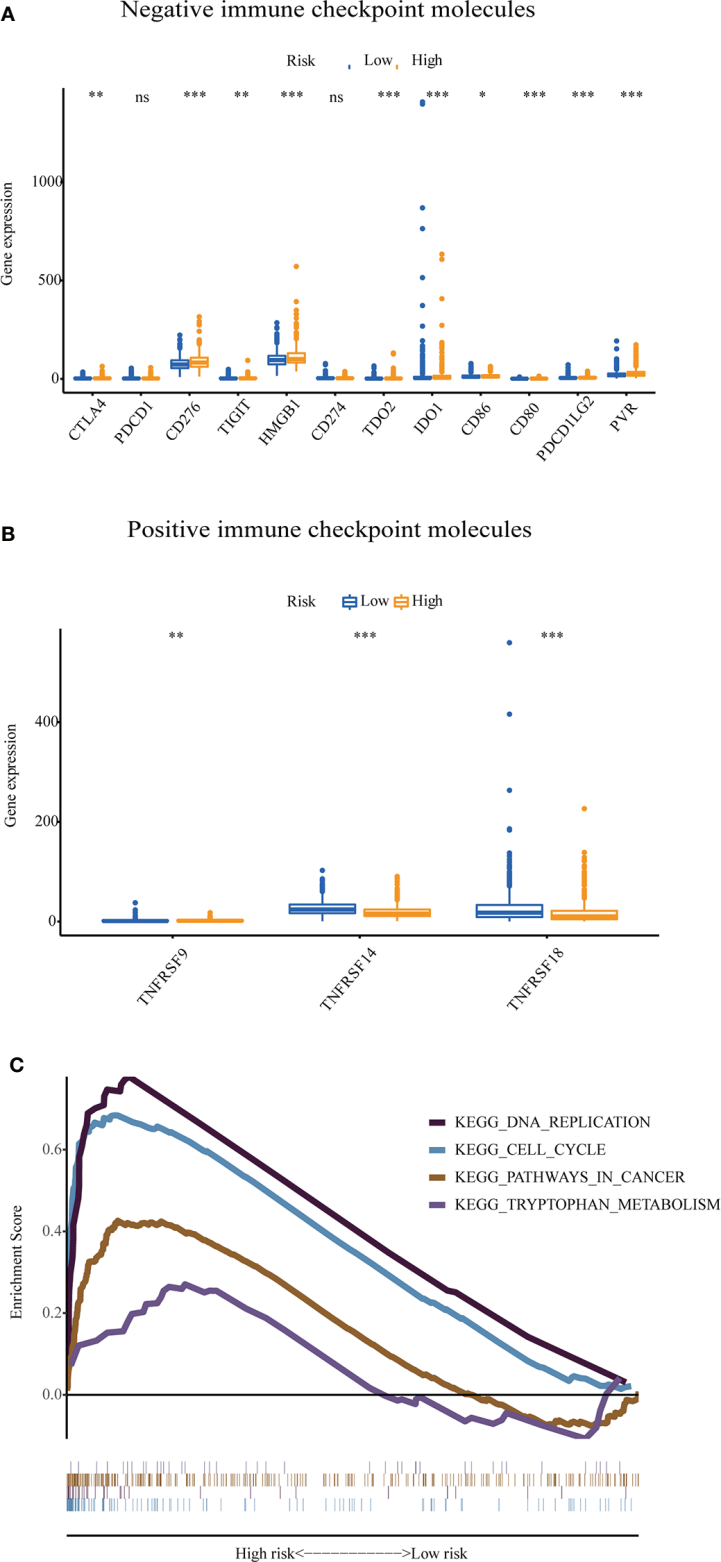
The expression level of some immune checkpoint molecules and representative transcriptome traits of biological function between the patients in the high-risk group and low-risk group. **(A)** The negative immune checkpoint molecules. **(B)** The positive immune checkpoint molecules. **(C)** Representative transcriptome traits of biological function in multiple GSEAs of patients in the high-risk group and low-risk group.GSEA, gene set enrichment analysis. *p < 0.05, **p < 0.01, ***p < 0.001, ns, no significance.

The GU-like and GS-like groups had distinct immune checkpoint expression levels, including CTLA4, IDO1, and TDO2. We next analyzed the association of the risk score with the expression of some immune checkpoint molecules in breast cancer. As shown in **Figure 9A**, the higher risk score was significantly associated with higher expression of some negative immune checkpoint molecules in breast cancer patients, including CTLA4, CD276, TIGIT, PVR, HMGB1, TDO2, IDO1, CXCL9, and CXCL10. However, there was no link between the risk score and PDCD1 (PD-1) or CD274 (PD-L1) expression. As shown in **Figure 9B**, the lower-risk score was positively associated with the expression of positive immune checkpoint molecules, including tumor necrosis factor receptor superfamily (TNFRSF) 9, TNFRSF14, and TNFRSF18. Multiple GSEAs indicated that the group with a high-risk score was enriched in DNA replication (NES = 1.893, *p*< 0.01), cell cycle (NES = 2.077, *p<* 0.001), pathways in cancer (NES = 1.631, *p<* 0.05), and tryptophan metabolism (NES = 1.560, *p*< 0.05) (**Figure 9C**).

## Discussion

Breast cancer pathogenesis partly originated from GI. Anti-HER2 therapy has improved the survival rate for patients with HER2 amplification (4, 28). Although the improvement of early detection and treatment has decreased the death rate for breast cancer patients during the past decades, almost all metastatic patients eventually succumb to death (29). Currently, single-cell approaches and high-throughput multicellular sequencing technologies can detect genetic alteration for cancer patients (30), but the degree of GI still needs to be explored. NORAD has been proven to be indispensable for keeping GI (14, 15), indicating a close association of lncRNA and GI.

This study determined 128 GI-related lncRNAs using somatic mutation and lncRNA expression data of breast cancer patients. After analyzing the co-expressed genes, we found that the expression levels of some negative immune checkpoints, including CTLA4, IDO1, and TDO2, were closely associated with these lncRNAs. Furthermore, the functional analysis suggested that the 1,280 co-expressed genes were mainly enriched in the MAPK signaling pathway. It has been reported that the MAPK pathway participates in regulating cell differentiation, proliferation, survival, and death and is considered as the most frequently mutated pathway in cancer patients (31). Using univariate and multivariable Cox proportional hazards regression analysis, we generated the prognostic model of six lncRNAs, including U62317.4, MAPT-AS1, AC115837.2, EGOT, SEMA3B-AS1, and HOTAIR. The model was proved to predict survival independently from other clinical features, including gender, age, and stage.

According to the coefficient of each lncRNA, we found that HOTAIR and AC115837.2 increased the risk score of a breast cancer patient, while U62317.4, EGOT, MAPT-AS1, and SEMA3B-AS1 tended to decrease the score. The survival analysis in GEPIA demonstrated that high expression of EGOT, MAPT-AS1, and SEMA3B-AS1 predicted favorable OS, which was consistent with their coefficients. Xu et al. reported that low level of EGOT expression was associated with poor OS (32). A previous study showed that HORAIR was overexpressed in primary and metastatic breast cancer patients, which could predict the possibility of metastasis and death (31). In contrast to the lncRNAs above, the function of AC115837.2 remains not clear yet. For U62317.4, it was suggested to be an autophagy‐related lncRNA and was included in prognosis-related risk models in breast cancer and bladder cancer (33, 34). Qiu et al. demonstrated that EGOT was lowly expressed in cell lines and breast cancer tissues and may suppress cell migration and viability (35). MAPT-AS1 exists at the anti-sense strand of the MAPT promoter region. Pan et al. indicated that reducing the expression of MAPT-AS1 restrained the migration and proliferation of ER^-^ breast cancer cells (36). It has been reported that SEMA3B-AS1 was deemed as a novel cancer suppressor in gastric cardia adenocarcinoma, esophageal squamous cell carcinoma, and hepatocellular carcinoma (37–39). Moreover, Li et al. suggested that SEMA3B-AS1could be used as part of the stemness-associated lncRNA prognostic signature in breast cancer (27).

According to the prognostic model in this study, the breast cancer patients in the training set could be divided into two groups with high or low risk, indicating an utterly different OS and somatic mutation level. Moreover, the result has been validated on the independent testing set and the TCGA set. Most importantly, the prognostic model could be applied on breast cancer patients with any age and pathologic stages. However, there was a significant association between the low-risk score and favorable OS in female patients rather than male patients, probably due to the insufficient number of male patients. The ROC area of OS showed that our prognostic model was superior to the other existing two models in breast cancer. Four lncRNAs in our model were covered in GEPIA and survival analysis showed that they were closely related with OS. These validation results demonstrated that our prognostic model may predict prognosis of breast cancer. Additionally, drugs that target certain aberrantly expressed genes or non-coding RNAs show a more potent anticancer efficiency and lower toxicity than conventional chemotherapies (40). Thus, the six lncRNAs may serve as potential therapeutic targets.

Nowadays, cancer immunology and immunotherapy provide a novel perspective for cancer therapeutics (41). Cancer immune escape mechanism is considered a potential target in cancer immunotherapy (42, 43). Therefore, we analyzed some immune checkpoint molecules between the high- and low-risk groups. The result implied that the patients with a high risk had higher expression of negative immune checkpoints, such as CTLA4, CD80, CD86, IDO1, and TDO2. CTLA-4 on T cells binds to B7 molecules (CD80 and CD86) on the antigen-presenting cells, blocking co-stimulation and then terminating T-cell activation (44–46). Tryptophan catabolism has a pivotal role in forming immune evasion and immune tolerance (47). Both IDO1 and TDO2 are rate-limiting enzymes in tryptophan degradation. With ICIs becoming a powerful new strategy for cancer therapy, it is necessary to identify patients who are suitable for ICIs to avoid severe immune-related adverse effects (irAEs), especially autoimmune diseases. A systematic review reported that irAEs could occur in any organ and impact 89% of patients treated with CTLA-4 inhibitors and 74% of those receiving PD-1/PD-L1 inhibitors (48). Thereinto, ICI-induced endocrinopathies are the most common irAEs, which are presumed to result in permanent, irreversible endocrine dysfunction (49). Though transient inflammation affecting most systems resolves with steroid therapy and is followed by restoration of normal organ function, administering ICIs to patients should still be deliberately considered. This model may predict ICI efficacy, facilitating the identification of patients who are responsive to ICIs precisely. Therefore, patients potentially benefiting from ICIs can be screened out and needless irAEs are avoided as the unresponsive population has been excluded.

Furthermore, the multiple GSEA results suggested that the breast cancer patients with a high risk were mainly associated with genes involved in DNA replication, cell cycle, pathways in cancer, and tryptophan metabolism. This suggested that there tends to be a rapid progression in breast cancer patients with a high risk. Meanwhile, the patients with a low risk had more positive immune checkpoints, such as TNFRSF9, TNFRSF14, and TNFRSF18. The proliferation of antigen-primed CD8^+^ T cells could be stimulated by the interaction between the tumor necrosis factor ligand and cognate TNFRSF, which is beneficial for protective immunity and cancer immunotherapy (50).

## Conclusion

To sum up, we constructed a GI-related prognostic risk model comprising six lncRNAs (U62317.4, MAPT-AS1, AC115837.2, EGOT, SEMA3B-AS1, and HOTAIR) in breast cancer. This model may have improved predictive value compared to other existing models and provide novel therapeutic opportunities for breast cancer patients.

## Data availability statement

The original contributions presented in the study are included in the article/**Supplementary Material**. Further inquiries can be directed to the corresponding author.

## Author contributions

YJ collected data, prepared the figures, and drafted the manuscript. SL prepared the figures, organized the structure, and checked the manuscript. XW conducted the real-time PCR assay. LZ, MY, HW, SR, and KZ participated in the discussion. All authors contributed to the article and approved the submitted version.

## Funding

This work was supported by the National Natural Science Foundation of China (No. 82172825) and the Chinese Society of Clinical Oncology Foundation (No. Y-BMS2019-070).

## Acknowledgments

All authors appreciate TCGA (https://portal.gdc.cancer.gov/) databases for offering the data on breast cancer.

## Conflict of interest

The authors declare that the research was conducted in the absence of any commercial or financial relationships that could be construed as a potential conflict of interest.

## Publisher’s note

All claims expressed in this article are solely those of the authors and do not necessarily represent those of their affiliated organizations, or those of the publisher, the editors and the reviewers. Any product that may be evaluated in this article, or claim that may be made by its manufacturer, is not guaranteed or endorsed by the publisher.

## Supplementary material

Supplementary Figure 1 Mutation landscape of breast cancer patients with a high **(A)** or low **(B)** risk. Del, deletion; Ins, insertion; OS, overall survival.

Supplementary Figure 2 The prognostic value of 6-GI-related lncRNA prognostic model in breast cancer patients with distinct clinical features. **(A)** age > 65; **(B)** age ≤ 65; **(C)** female; **(D)** male; **(E)** stage I-II; **(F)** stage III-IV.

Supplementary Figure 3 The prognostic value of the lncRNA-related model in breast cancer patients with different T, N, or M stages. **(A)** T1-2, **(B)** T3-4, **(C)** N0, **(D)** N1-3, **(E)** M0, and **(F)** M1.

Supplementary Figure 4 The ROC analyses for 3-year **(A)** and 5-year **(B)** overall survival for the JiaolncSig, LilncSig, and LiulncRNA.

## Glossary

AUC: area under ROC curve
CI: confidence interval
CIBERSORT: Cell-type Identification by Estimating Relative Subsets of RNA Transcripts
CTLA-4: cytotoxic T-lymphocyte-associated protein 4
EGOT: glutathione reductase and glutamic oxaloacetic transaminase
ER: estrogen receptor
dMMR: deficient mismatch repair
FDR: false discovery rate
GEPIA: gene expression profiling interactive analysis
GI: genomic instability
GO: gene ontology
GS: genomically stable group
GSEA: gene set enrichment analysis
GU: genomically unstable
HER2: human epidermal growth factor receptor 2
HR: hazard ratio
HOTAIR: HOX transcript antisense RNA
ICI: immune checkpoint inhibitor
IDO1: indoleamine 2, 3-dioxygenase 1
KEGG: Kyoto Encyclopedia of Genes and Genomes
lncRNA: long non-coding RNA
MAPT-AS1: MAPT antisense RNA 1
MSI-H: microsatellite instability-high
NORAD: non-coding RNA activated by DNA damage
OS: overall survival
PD-1 (PDCD1): programmed cell death 1
PD-L1: programmed cell death receptor ligand 1
ROC: receiver operating characteristic
SEMA3B-AS1: Semaphorin 3B antisense RNA 1
TCGA: the cancer genome atlas
TDO2: tryptophan 2,3-dioxygenase 2
TNFRSF: tumor necrosis factor receptor superfamily.
