# Supplementary figures and images for: A genomic instability-related lncRNA model for predicting prognosis and immune checkpoint inhibitor efficacy in breast cancer
Source: Front Immunol. 2022 Aug 5;13:929846. doi: 10.3389/fimmu.2022.929846 (PMC9389369; doi:10.3389/fimmu.2022.929846)

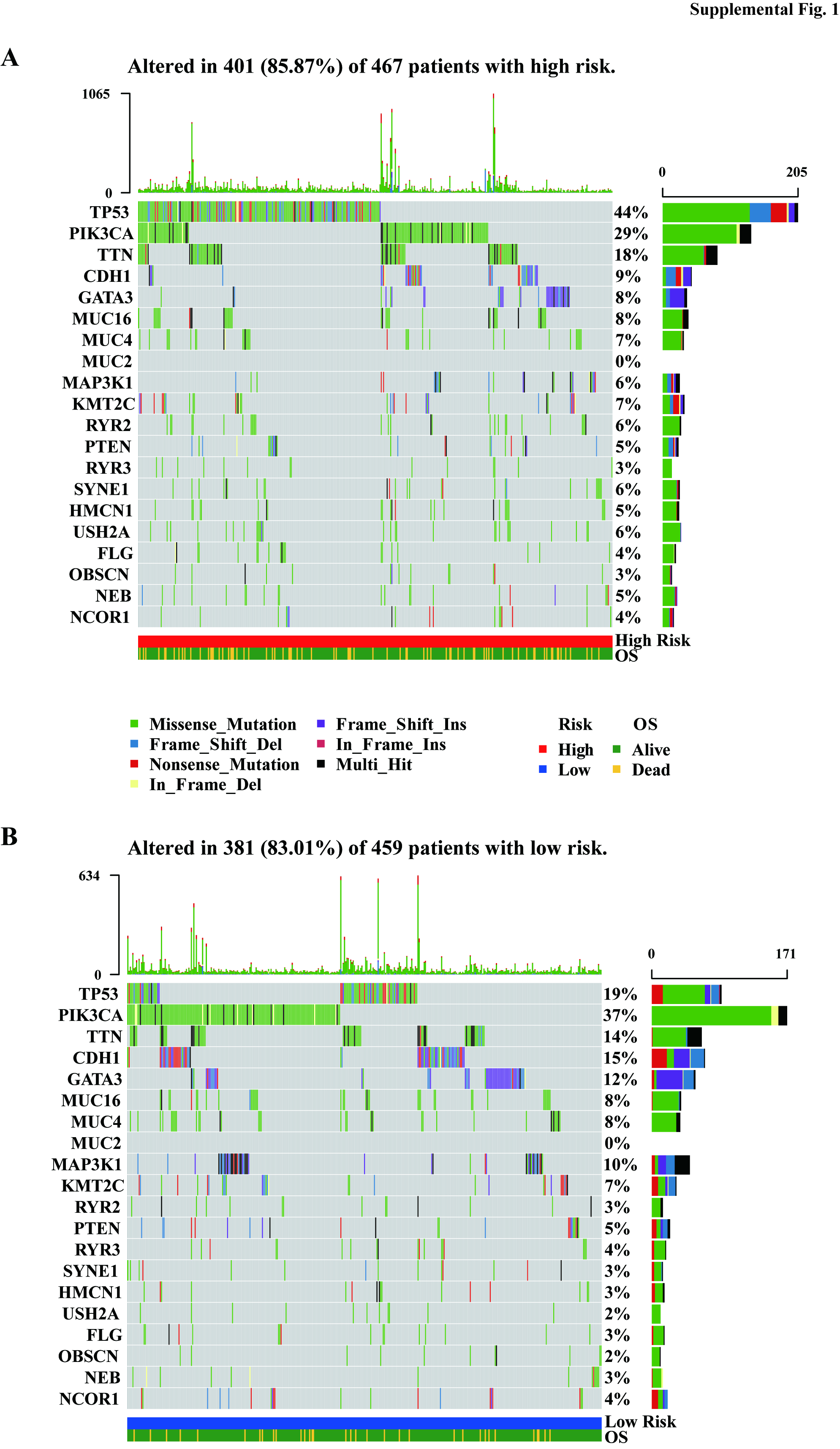

Supplement: Supplementary Figure 1 — Mutation landscape of breast cancer patients with a high (A) or low (B) risk. Del, deletion; Ins, insertion; OS, overall survival. [file Image_1.tif]

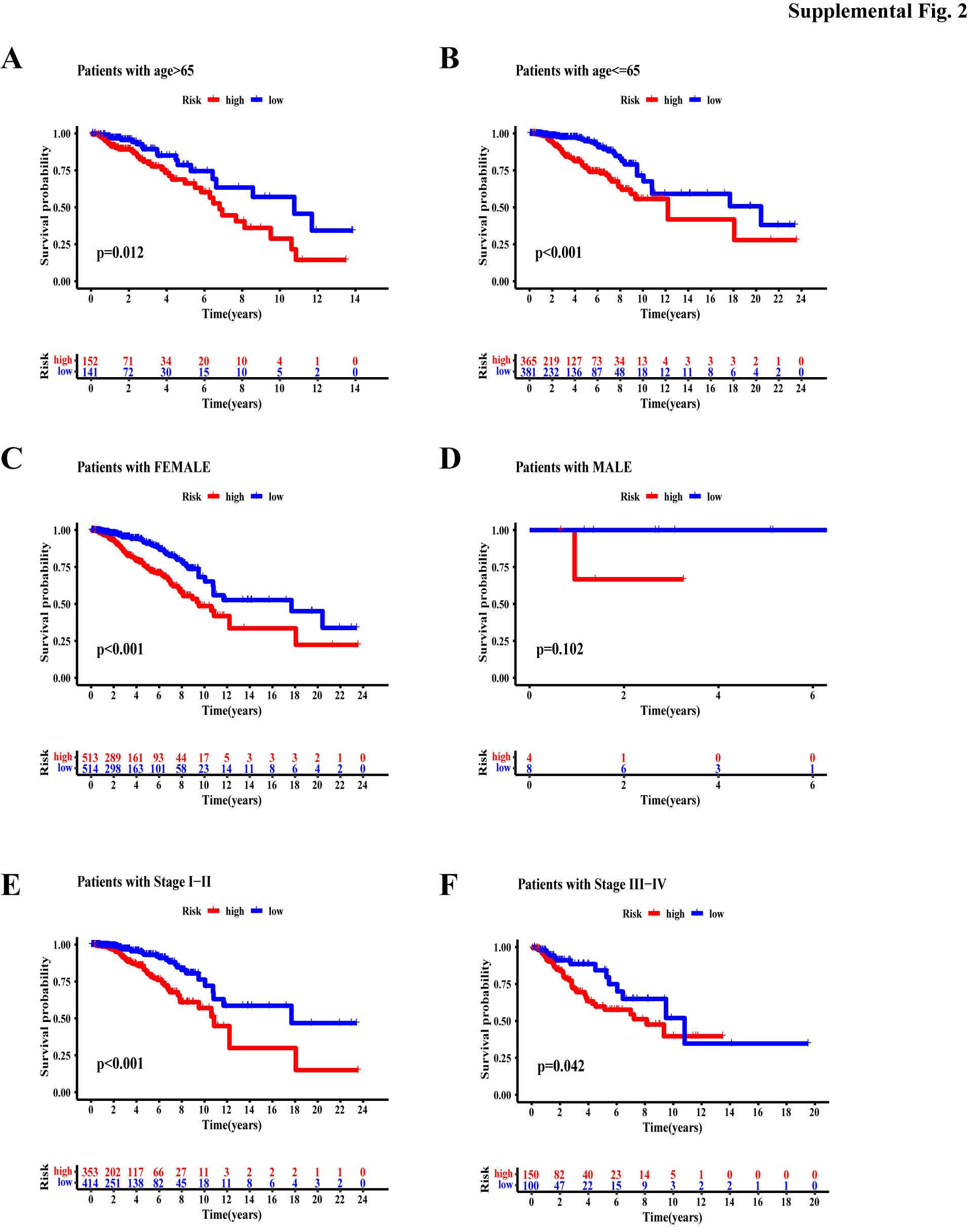

Supplement: Supplementary Figure 2 — The prognostic value of 6-GI-related lncRNA prognostic model in breast cancer patients with distinct clinical features. (A) age > 65; (B) age ≤ 65; (C) female; (D) male; (E) stage I-II; (F) stage III-IV. [file Image_2.tif]

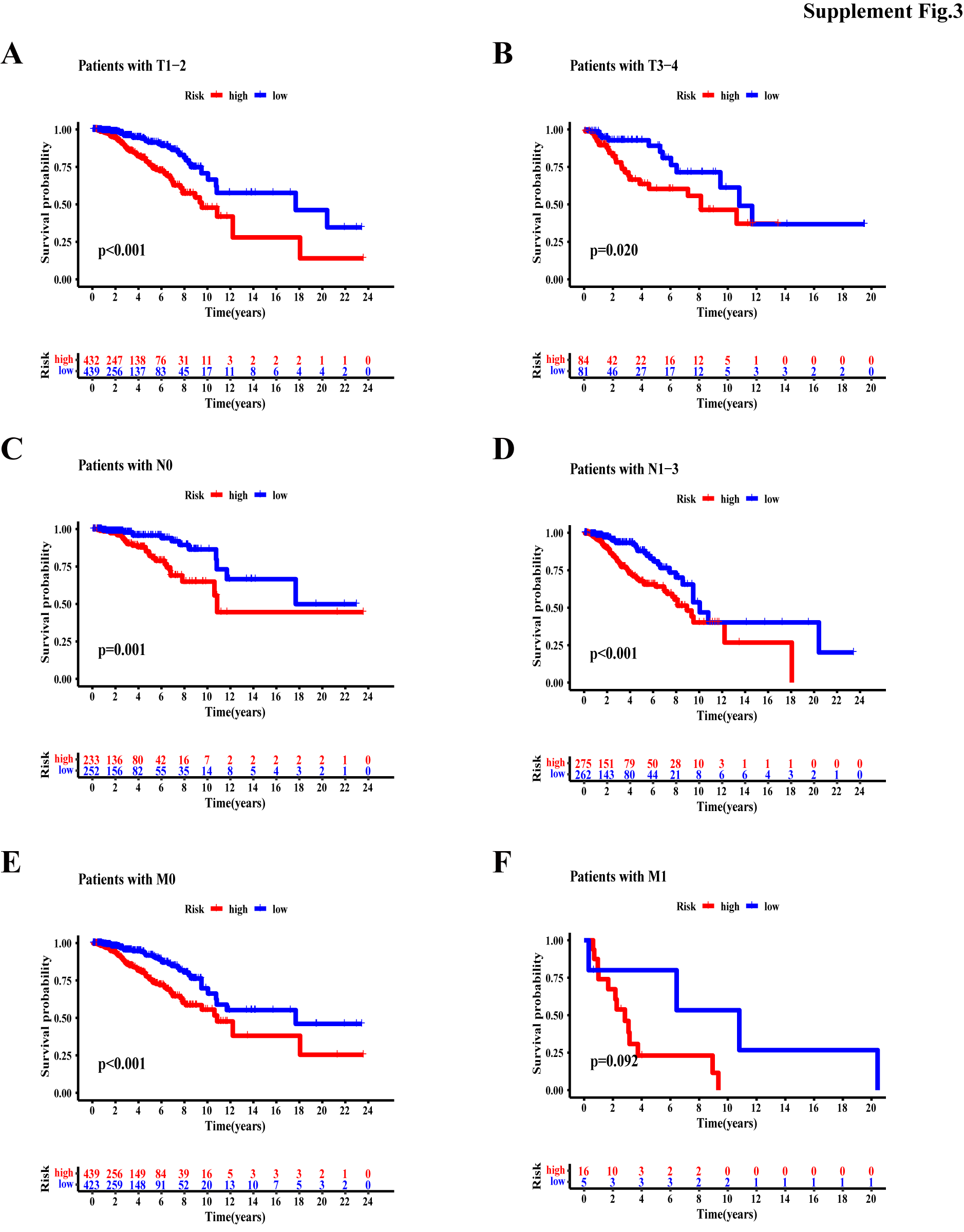

Supplement: Supplementary Figure 3 — The prognostic value of the lncRNA-related model in breast cancer patients with different T, N, or M stages. (A) T1-2, (B) T3-4, (C) N0, (D) N1-3, (E) M0, and (F) M1. [file Image_3.tif]

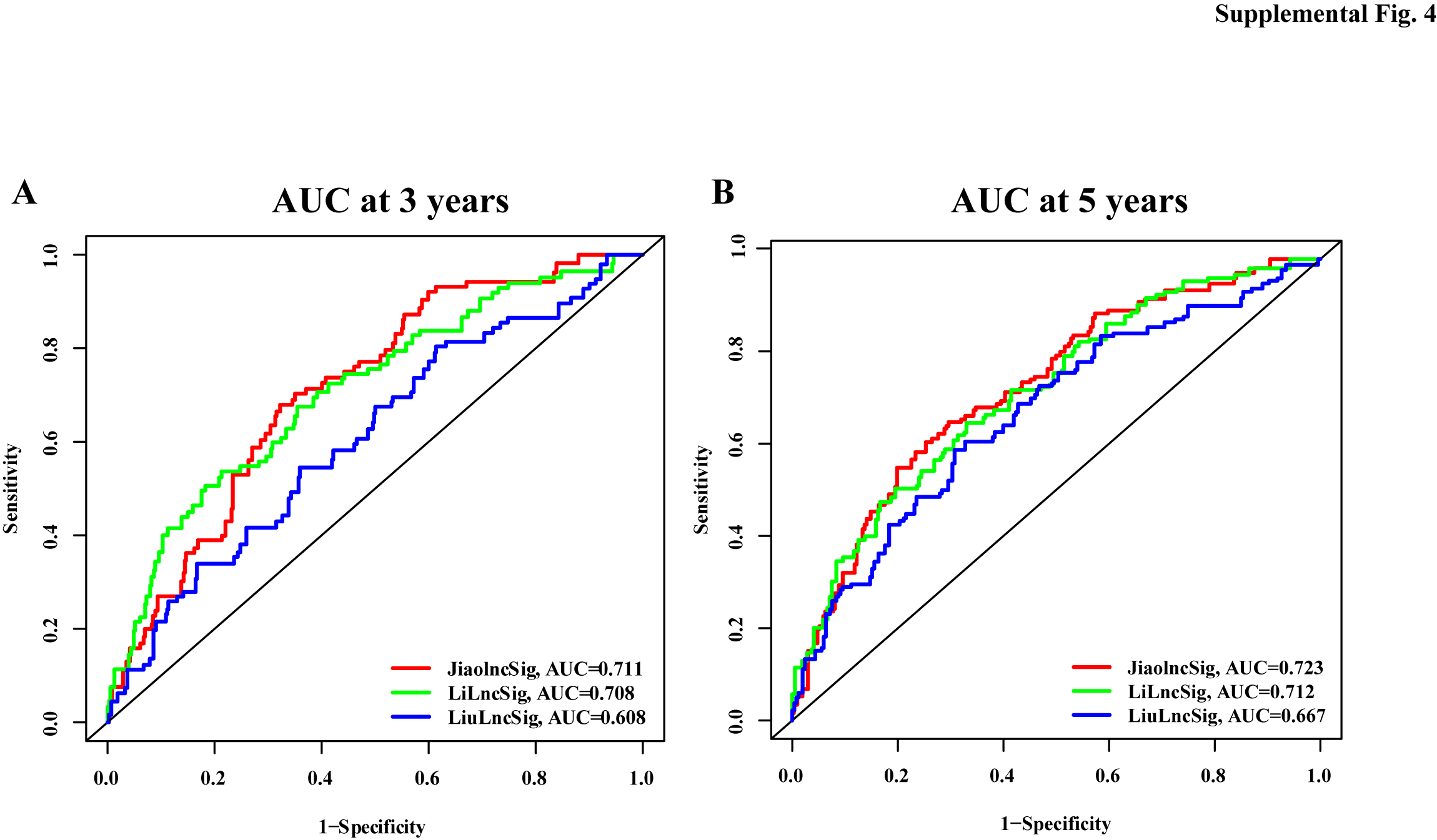

Supplement: Supplementary Figure 4 — The ROC analyses for 3-year (A) and 5-year (B) overall survival for the JiaolncSig, LilncSig, and LiulncRNA. [file Image_4.tif]
